# Heterogeneity of immune cells and their communications unveiled by transcriptome profiling in acute inflammatory lung injury

**DOI:** 10.3389/fimmu.2024.1382449

**Published:** 2024-04-30

**Authors:** Zhi-ying Kang, Qian-yu Huang, Ning-xin Zhen, Nan-xia Xuan, Qi-chao Zhou, Jie Zhao, Wei Cui, Zhao-cai Zhang, Bao-ping Tian

**Affiliations:** ^1^ Department of Critical Care Medicine, The Second Affiliated Hospital, Zhejiang University School of Medicine, Hangzhou, Zhejiang, China; ^2^ Department of Critical Care Medicine, The First Affiliated Hospital, Ningbo University, Ningbo, Zhejiang, China

**Keywords:** bulk RNA-seq, single-cell RNA-seq, ALI/ARDS, heterogeneity, immune cells, cell-cell communication

## Abstract

**Background:**

Acute Respiratory Distress Syndrome (ARDS) or its earlier stage Acute lung injury (ALI), is a worldwide health concern that jeopardizes human well-being. Currently, the treatment strategies to mitigate the incidence and mortality of ARDS are severely restricted. This limitation can be attributed, at least in part, to the substantial variations in immunity observed in individuals with this syndrome.

**Methods:**

Bulk and single cell RNA sequencing from ALI mice and single cell RNA sequencing from ARDS patients were analyzed. We utilized the Seurat program package in R and cellmarker 2.0 to cluster and annotate the data. The differential, enrichment, protein interaction, and cell-cell communication analysis were conducted.

**Results:**

The mice with ALI caused by pulmonary and extrapulmonary factors demonstrated differential expression including Clec4e, Retnlg, S100a9, Coro1a, and Lars2. We have determined that inflammatory factors have a greater significance in extrapulmonary ALI, while multiple pathways collaborate in the development of pulmonary ALI. Clustering analysis revealed significant heterogeneity in the relative abundance of immune cells in different ALI models. The autocrine action of neutrophils plays a crucial role in pulmonary ALI. Additionally, there was a significant increase in signaling intensity between B cells and M1 macrophages, NKT cells and M1 macrophages in extrapulmonary ALI. The CXCL, CSF3 and MIF, TGFβ signaling pathways play a vital role in pulmonary and extrapulmonary ALI, respectively. Moreover, the analysis of human single-cell revealed DCs signaling to monocytes and neutrophils in COVID-19-associated ARDS is stronger compared to sepsis-related ARDS. In sepsis-related ARDS, CD8+ T and Th cells exhibit more prominent signaling to B-cell nucleated DCs. Meanwhile, both MIF and CXCL signaling pathways are specific to sepsis-related ARDS.

**Conclusion:**

This study has identified specific gene signatures and signaling pathways in animal models and human samples that facilitate the interaction between immune cells, which could be targeted therapeutically in ARDS patients of various etiologies.

## 1 Introduction

Currently, the standard treatment for ARDS includes supportive care measures such as mechanical ventilation, fluid management, and infection control. While these interventions are important, they do not address the underlying causes of ARDS or consider the individual differences between patients, and a well-established precision treatment strategy to effectively address the various damage caused by ARDS has yet to be proposed (1–3). Therefore, the incidence and mortality rate of ARDS remains high in critically ill patients (4).The heterogeneity can at least partially explain the diversity of manifestations in ARDS patients and the variability in treatment outcomes (5, 6). For instance, the second analysis of the clinical trials have identified distinct subphenotypes of ARDS, each with different clinical outcomes, inflammatory profiles, and responses to therapies, highlighting the importance for a further understanding of heterogeneity in ARDS (7–14). The response of the immune system plays a crucial role in the development of ARDS, which is closely associated with pulmonary inflammation, and immune regulation can modulate the intensity and duration of the inflammatory response, preventing excessive inflammation and the development of inflammatory storms. By inhibiting the production of inflammatory mediators and regulating the activity and movement of inflammatory cells, immune regulation can alleviate the inflammatory response in ARDS. Furthermore, immune cells such as alveolar macrophages, neutrophils, and lymphocytes also play significant roles in ARDS (15–17). Immune regulation can modulate the quantity, activity, and function of these immune cells, thereby reducing immune cell-mediated lung injury. Additionally, the repair of lung tissue is a critical process in restoring lung function after ARDS. Immune regulation can promote the repair and regeneration of lung tissue while minimizing fibrosis and scar formation. For instance, by regulating the production of cytokines and the proliferation of cells, immune regulation can enhance lung tissue repair. In conclusion, immune regulation in ARDS is crucial in managing the inflammatory response, modulating the activity and function of immune cells, and facilitating lung tissue repair. These regulatory effects have significant implications for the treatment and prevention of ARDS. However, currently, we know very little about the differences in immune cells and their roles in ARDS caused by different reasons.

In this study, we explored the variability in gene expression patterns associated with acute lung injury (ALI). By analyzing bulk-RNA and scRNA data, we identified five genes, Clec4e, Retnlg, S100a9, Coro1a and Lars2, which play a role in identifying and treating different factors contributing to acute lung injury. Meanwhile, through the analysis of bulk RNA sequencing data, we found that inflammatory factor-related pathways play a great role in ALI caused by extra-pulmonary factors, whereas the pathways that play a role in ALI caused by pulmonary factors are more complex, and we also found that Tnf plays a great role in both subtypes of ALI. At the level of individual cells, we successfully discovered the involvement of MIF, CSF3, TGFβ and CXCL signaling pathways in facilitating communication between immune cells in cases of extra-pulmonary and pulmonary ALI, respectively. And the cell-cell communications that differed most in the two subtypes of ALI were found to be autocrine in neutrophils and B cells, respectively, as well as B cells and NKT cells on M1 macrophages. Through the analysis of single-cell sequencing data from human peripheral blood, we have identified the substantial role of dendritic cells in COVID-19-associated ARDS. Furthermore, CD8+ T cells and Th cells were found to be involved in sepsis-related ARDS, along with the MIF and CXCL signaling pathways in sepsis-related ARDS. These findings indicate that targeting specific gene signatures or pathways could potentially serve as an effective strategy for preventing various subtypes of ARDS.

## 2 Methods

### 2.1 Ethics statement

The animal experiments were approved by the Ethics Committee of the Second Affiliated Hospital, Zhejiang University School of Medicine (No.2022050).

### 2.2 Animals

Six to eight weeks old male C57BL/6 mice of SPF grade were purchased from Beijing Vital River Laboratory Animal Technology Co., Ltd. The mice were fed in the standard animal laboratory at 25 ± 1°C with humidity of 60 ± 5% and 12-hous light/dark cycle, adequate food and water were supplied. The mice were allowed to acclimated to the environment for 1 week prior to the experiment. The mice were randomly divided into control, pulmonary ALI, and extra-pulmonary ALI group. Pulmonary ALI was induced by intratracheal injection of Lipopolysaccharide (LPS) (10 mg/kg, from Pseudomonas aeruginosa 27316, Sigma-Aldrich, USA, L9143) dissolved in 50 μl sterile PBS. Additionally, we used intraperitoneal injection of LPS (25 mg/kg) to construct sepsis-related ARDS, known as extra-pulmonary ALI (18). The mice enjoyed free access to food and water and were monitored until sacrificed 24 h after the treatment. Lung tissue specimens were collected for subsequent testing and analysis.

### 2.3 Bulk RNA sequencing

Extract total RNA from lung tissue, assess the quality of the RNA samples, and then proceed with the bulk RNA sequencing. Transcriptome library was constructed using mRNA sourced from lung tissue of both control, pulmonary and extra-pulmonary group. Sequencing was performed on the BGISEQ platform, yielding raw reads. These raw reads underwent a filtration process to remove low-quality reads, adapter contaminants, and reads containing unknown base N content. Subsequently, the remaining high-quality reads were aligned to the reference genome sequence (GCF_000001635.26_ GRCm38.p6) available at the National Center for Biotechnology Information (NCBI) using the Bowtie2 software (19, 20).

### 2.4 Identify the differential expressed genes

After preprocessing the RNA-seq data using the aforementioned steps, the counts can be used for normalization. Subsequently, the DESeq2 software package can be employed to identify differentially expressed genes (DEGs) and calculate their statistical significance (21). The select criteria of DEGs were set as Bonferroni-adjusted p-value < 0.05 and |log2 FC| > 1,5, where log2 FC > 1.5 indicated upregulated genes and log2 FC < -1.5 indicated downregulated genes.

### 2.5 Functional enrichment analysis of DEGs

The selected differentially expressed genes were imported into the David for Gene Ontology (GO) and Kyoto Encyclopedia of Genes and Genomes (KEGG) enrichment analysis (22). Genes with log2 FC > 1 were defined as upregulated, and those with log2 FC < 1 were defined as downregulated. The top 20 pathways with the highest significance (P < 0.05) and the largest fold change (FC) were selected, and a bubble chart was created to show the enrichment of upregulated and downregulated pathways (23).

### 2.6 Gene set variation analysis

The sequencing data were subjected to gene set variation analysis (GSVA) using R software. The top 20 pathways with the largest differences were selected for visualization based on p-values. GSVA, heatmap, and bubble chart were generated using packages such as gseaplot2, enrichplot, ggplot2, and heatmap (24).

### 2.7 Preparation of single-cell suspension from lung tissue

The collected mouse lung tissue was centrifuged to remove the tissue preservation solution, washed twice with DMEM, and then quickly cut into 2-3 mm-sized pieces before being digested at 37°C. Staining the cells with Trypan Blue for cell counting. Cell digestion was terminated when large tissue were unobserved. The resulting cell suspension was filtered through a 40 µm cell sieve, followed by centrifugation at 300 g for 5 min at 4°C, and subsequent discarding of the supernatant. Red Blood Cell Lysis Buffer was then added, and the lysis process was concluded at the situation of 0.04% BSA in PBS before another round of centrifugation and removal of the supernatant. The cells were washed and resuspended in 0.04% BSA in PBS, then the cells were dyed with Trypan Blue once again. The viability of the cells was assessed using a hemocytometer. Cells with a viability exceeding 90% were utilized for subsequent analyses.

### 2.8 10×genomics single-cell RNA sequencing

Lung single-cell suspensions underwent processing via a fully automated instrument known as the Chromium Controller. This instrument utilized a microfluidic system to sort the cells. Barcode-containing Gel Beads were employed to bind to the cells, forming unique single-cell GEMs (Gel Bead in Emulsion) structures. Upon cell lysis, mRNA was released, subsequently reverse transcribed to generate double-stranded cDNA. The GEMs underwent emulsion breaking, leading to the construction of the library via PCR using the cDNA. The library construction followed the protocol CG000204_ChromiumNextGEMSingleCell3_v 3.1_Rev_D, with library concentration determined using the Qubit method and fragment size assessed using Caliper. Library quality control criteria included a library concentration exceeding 3 ng/μl and the main peak of the library fragment measuring 300-600 bp in size. Following successful library quality control assessment, the single-stranded cyclic DNA molecules underwent sequencing via rolled-loop replication and probe-anchored polymerization technology.

### 2.9 Single-cell data preprocessing

Cell Ranger was used to process the raw data and generate the raw unique molecular identifier (UMI) count matrix. The merged matrix was transferred into the R statistical environment for further analysis using the Seurat package (v.4.3.0.1). Quality testing of the mouse data revealed that the data scores were closer to 1 (**Supplementary Figure S1A**). The cells with mitochondrial UMI counts greater than 10%, or below 200 genes were identified as low-quality cells and removed (**Supplementary Figure S1B**). Batch effects were corrected by the “SCT” function. Principal component analysis (PCA) was performed to reduce the dimension of the scRNA-Seq dataset. We used TOP 30 PCs for downstream analysis. The cells were clustered and identified by the “FindNeighbors” and “FindClusters” functions with a resolution of 0.3. The clusters were projected into a two-dimensional plot for visualization using the “RunUMAP” functions (25–27).

### 2.10 Cluster marker identification and cell type annotation

The main reference data for cell marker genes used for cellular annotation were obtained from CellMarker 2.0. Firstly, all cells were divided into 16 major cell types, and then after cell subpopulation annotation, a statistical analysis was performed to assess the proportion of cells from different groups within each cell subpopulation (28).

### 2.11 Functional enrichment analysis

GO enrichment of DEGs of cell subgroups was performed using the “compareCluster” function in the “clusterProfiler” packages.

### 2.12 Cell-cell communication analysis

We used CellChat to infer cell–cell interactions between immune cell subsets (29), and cells with a total number greater than 200 were left for analysis. The potential interaction strength was predicted based on the expression of immune-associated senders and receptors. Build a CellChat object and import the ligand-receptor database for mice, “CellChatDB.mouse.” Preprocess the data, and then utilize the compute CommunProb algorithm to calculate the communication probability and infer cell-cell interaction networks (29). Use the computeCommunProbPathway algorithm to calculate cell communication networks at the ligand-receptor pair level and signaling pathway level. Finally, visualize the results using algorithms such as netVisual_aggregate, netVisual_circle, and netVisual_bubble. To reveal the differences in communication patterns between immune cells in the two groups, we chose MIF signaling pathway and CXCL signaling pathway with the greatest differential expression for further visualization.

### 2.13 Human single-cell data acquisition and analysis

Two sets of human peripheral blood single cell sequencing data, GSE150728 and GSE151263, were obtained from the Gene Expression Omnibus (GEO) database. The first three samples from the control group in GSE150728 were selected as the control group for this analysis, and the samples with concurrent ARDS in the sepsis group were selected as the sepsis-related ARDS group for this study. In this study, samples with COVID-19 in GSE151263 and admitted to the hospital with a diagnosis of ARDS were selected as the COVID-19-associated ARDS group. Genes expressed in less than 3 cells and cells with less than 200 genes were removed from the three groups, respectively, and the data from the three groups were combined and analyzed. Subsequent conditions were set as in mouse lung tissue.

### 2.14 Hematoxylin-eosin staining

Hematoxylin-eosin (HE) staining was performed to explore damage in the lung tissue. Prior to histological analysis, lungs were immersed in 4% paraformaldehyde for 24 hours. Paraffin-embedded sections (3 μm thickness) were prepared and subjected to HE staining. The sections were placed in aqueous hematoxylin solution for 3-5 minutes. Then, they were washed with running water for 1-2minutes, then dehydrated in alcohol for 1-2 minutes. Finally, they were treated with permeation and fixation. Light microscopy images were obtained using an microscope (Olympus, CX-31). The degree of congestion, edema, exudation, and tissue damage in the lung tissue could be evaluated.

### 2.15 Immunohistochemistry

Lung tissue sections were obtained using the same method as above. Tissue sections were stained with anti-MIF(Affinity, DF6406), anti-CSF3 (Bioss, bs-1023R), anti-CXCL3 (Affinity, DF8554) and anti-TGF-β (Affinity, BF8012) overnight at 4°C. Goat Anti-rabbit IgG (HRP) (Abcam, ab205718) and Goat Anti-mouse IgG (HRP) (Abcam, ab205719) were used as secondary antibody. After the final staining, the samples were scanned using fluorescence microscope (Olympus, CX-31) and processed by CaseViewer (2.4).

## 3 Results

### 3.1 The various causes of acute lung injury display differences in both disease characteristics and overall transcriptomic profiles

In this study, the hematoxylin and eosin histological staining revealed a significant abnormalities in blood vessels, leakage of red blood cells into the air sacs, and excess fluid in the surrounding tissues in extra-pulmonary ALI mice. In contrast, pulmonary ALI mice showed an accumulation of cells responsible for inflammation in the air sacs, damage to the protective layer of cells, collapse of the pulmonary alveolus, and thickening and disruption of a transparent membrane (**Supplementary Figure S2A**). In order to further differentiate the potential differences in ALI caused by different reasons, bulk RNA-sequence was conducted on lung tissues from control, pulmonary, and extra-pulmonary mice. Correlation analysis (**Supplementary Figure S2B**) indicated that the correlation coefficients between two out of the 15 samples were between 0.91 and 1, signifying high and positive correlations. After establishing the stability of the samples and the data, variability analysis was continued using principal component analysis to reduce the effect of consistency. The two principal components, Dim1 and Dim2, were used to indicate the main sources of variation in the dataset (**Supplementary Figure S2C**). As suggested by the axes, 40.68% of the total variance was observed in the Dim1 dimension, while 12.43% of the total variance was observed in the Dim2 dimension. There is a significant clustering of points in the control and pulmonary groups in the Dim1 dimension, whereas there is a more significant clustering of points in the extra-pulmonary group in the Dim2 dimension. Overall the three groups of samples in the graph indicated that the 15 samples have some consistency within the group, while there are more significant differences between the groups.

**Figure 1:**
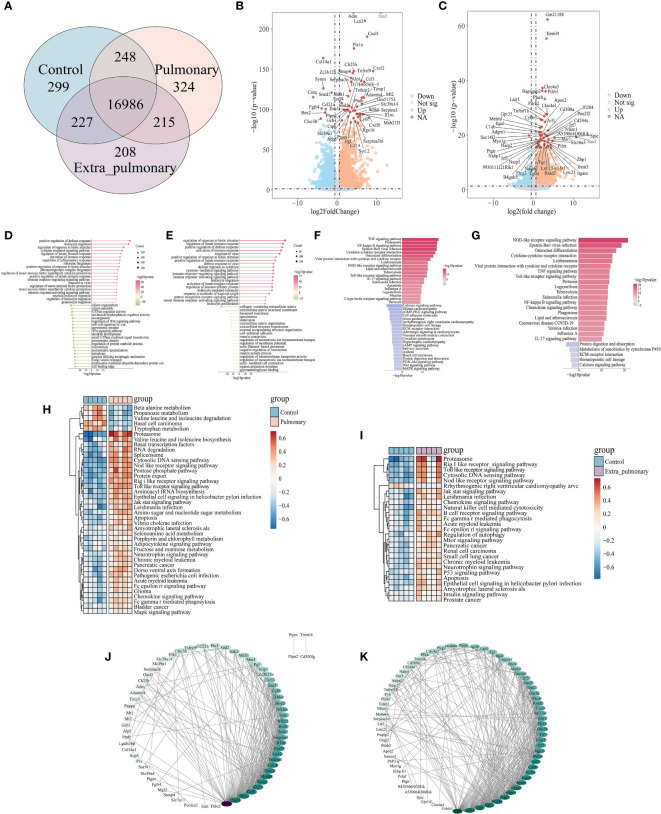
Analysis of bulk-RNA sequencing results showed significant differences in lung tissue between pulmonary and extra-pulmonary groups of acute lung injury mice. **(A)** Venn diagram of differential genes; **(B, C)** Volcano plot of differential genes in pulmonary and extra-pulmonary groups, orange represents log2FoldChange > 1, p-value < 0.05, blue represents log2FoldChange < -1, p-value < 0.05, and red represents differential genes in the top50; **(D, E)** Lollipop plot of top50 cell function as analyzed by GO enrichment in pulmonary and extra-pulmonary groups, pink represents increased expression and green represents decreased expression, and the size of the bubbles represents the number of genes; **(F, G)** Bar graph of KEGG enrichment analysis of top50 cellular pathway in pulmonary and extra-pulmonary groups, pink represents increasing expression and blue represents decreasing expression, and the closer the color to blue and pink represents the larger difference; **(H, I)** Heatmap of GSVA enrichment analysis of top50 cell pathway in pulmonary and extra-pulmonary groups, light blue indicates control group, light pink indicates pulmonary or extra-pulmonary group, brownish red represents increase in expression, blue represents decrease in expression, the closer the color is to blue and pink represents the bigger difference; **(J, K)** PPI network diagram of the top100 differential genes in pulmonary and extra-pulmonary group, with darker colors representing more critical genes for that gene.

Subsequently, we intersected the genes detected in the three groups and plotted the venn plot (**Figure 1A**), which showed that in addition to the 16,986 genes common to the three groups, there were still 787 genes in the pulmonary group and 650 genes in the extra-pulmonary group at this time, while only 215 genes common to the two groups were left, which showed that there were more different genes between the two groups. Next, we have identified the differential genes in the pulmonary and the extra-pulmonary compared with the control group, plotted the volcano diagram to show the top50 differential genes (**Figures 1B, C**). It can be seen that the differential genes of the two groups showed the phenomenon of increased expression over decreased expression in the overall distribution of genes in both groups, and this difference was more obvious in the extra-pulmonary group. And the differential genes in the top50 of both groups are more concentrated on one side of the orange scatter, indicating that the 50 genes with the largest differences in each of the two are also dominated by increased expression. After comparing the top50 genes of the two groups, it can be found that although the gene expression trends of the two groups showed similar changes, only the gene named Saa3 was present in the top50 differential genes of both groups. Differential gene enrichment of the two was analyzed and their top20 cellular functions and molecular pathways were demonstrated through information provided by Gene Ontology (GO) and Kyoto Encyclopedia of Genes and Genomes (KEGG) (**Figures 1D-G**). Although the two are roughly the same in terms of the type and number of cellular functions and signaling pathways activated, the number and type of genes in each function and signaling pathway, as well as the degree of activation of the function and pathway, are significantly different. For example, in the GO enrichment analysis, the leukocyte-related physiological functions became more active in the extra-pulmonary group, while ribosome-related biological functions were more pronounced in the pulmonary than in the extra-pulmonary group. Also in the KEGG enrichment analysis, NOD-like receptor signaling pathway, Toll-like receptor signaling pathway, Cytokine-cytokine receptor interaction, Viral protein interaction with cytokine and cytokine receptor, Chemokine signaling pathway and IL-17 signaling pathway, and other inflammation-related pathways had more pronounced activation in the extra-pulmonary group, whereas among the top20 of the pulmonary group only TNF signaling pathway, Proteasome and NF-κB signaling pathway had slightly higher activation than in the extra-pulmonary group. The same result was also observed in the heatmap of GSVA enrichment for differential genes (**Figure 1H, I**). In addition, GSVA also provided inflammation-related signaling pathways such as Rig I like receptor signaling pathway, Jak stat signaling pathway and Natural killer cell mediated cytotoxicity for subsequent studies. Finally we have analyzed the top100 differential genes for interactions using the STRING database and cytoscape, and mapped the network according to the density of their interactions (**Figures 1J, K**). It can be seen that the number of interactions between genes in the extra-pulmonary group is higher and denser relative to the -pulmonary group, but for the key gene Tnf common to both, it plays a more critical role in the pulmonary group.

The above bulk RNA-seq results suggest that the pathological processes of acute lung injury caused by different etiological factors are very different. For acute lung injury caused by systemic factors, the inflammation-related signaling pathways are more obviously activated, whereas lung injury caused by intrapulmonary factors, although the activation of inflammatory pathways is lower than that of extra-pulmonary groups, shows a more diversified and mixed activation of signaling pathways in general.

### 3.2 scRNA-seq analysis indicates notable variances in immune cell populations between the pulmonary and extra-pulmonary acute lung injury

**Figure 2:**
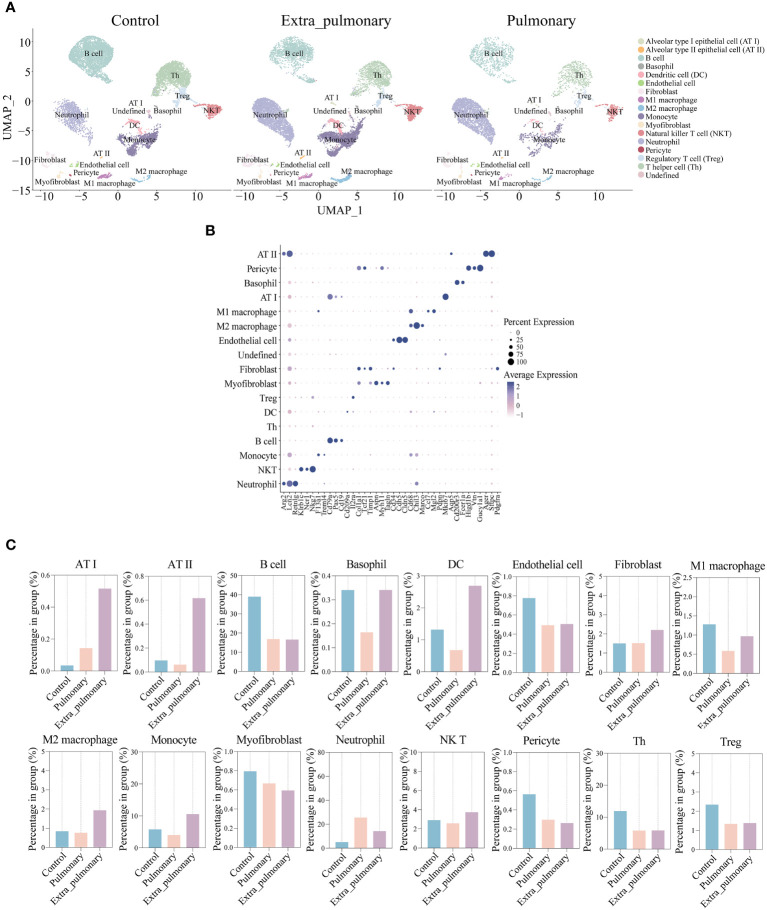
The single-cell data demonstrated significant differences in the cellular composition of the lung tissue of acute lung injury mice in the pulmonary and extrapulmonary groups. **(A)** UMAP dimensionality reduction plots of three groups of mouse lung tissue cells after clustering; **(B)** Bubble plot of marker gene expression in each population of cells, size represents how many cells express the gene, closer to blue indicates higher average expression; **(C)** Histograms of the percentage of cells in each of the three groups within their group, blue for the control group, light pink for the pulmonary group, and purple for the extra-pulmonary group.

Given that high-throughput sequencing can only identify alterations in overall gene expression and pathways in animal tissues, rather than offering specific insights for individual cells, we opted to perform single-cell sequencing and analysis on two mouse models of ALI induced by distinct pathogenic factors. In this study, single-cell sequencing was performed using the 10X Genomic platform. After standard data processing, low-quality cells were removed, resulting in a final dataset of 27,179 lung cells. This dataset consisted of cells from different groups, including 114,52 cells from the control group, 4871 cells from pulmonary acute lung injury (ALI), and 10,856 cells from extra-pulmonary ALI (**Supplementary Figure S3**). In order to clearly differentiate the cellular composition of the data, we sorted the genes in the mouse lung tissue experimental data in the cellmarker 2.0 database, which were used to categorize the cells, distinguishing between Alveolar type I epithelial cell (AT I), Alveolar type II epithelial cell (AT II), B cell, Basophil, Dendritic cell (DC), Endothelial cell, Fibroblast, M1 macrophage, M2 macrophage, Monocyte, Myofibroblast, Natural killer T cell (NKT), Neutrophil, Pericyte, Regulatory T cell (Treg) and T helper cell (Th), a total of sixteen types of cells, and the categorized data were presented by UMAP dimensionality reduction (**Figure 2A**). Each cluster of cells in the three groups are separated from each other, while each of their clusters is individually and agglomerately distributed, indicating a better quality of data filtering. In addition, we also selected a small number of marker genes for classification to be displayed to verify the accuracy of this classification method (**Figure 2B**). The origin of each gene it represents in the graph is the closest in color to blue at its corresponding cell, and the dots have the largest morphology, proving that the expression of different marker genes has its specific expression in each cell group species, and that the results of cell clustering are accurate and reproducible. The percentage of cells in each cluster among all cells in their respective groupings were calculated and plotted the histograms (**Figure 2C**). It can be found that in the pulmonary group B cells, neutrophils and Th cells were more predominant, while in the extra-pulmonary group B cells, neutrophils and monocytes were more predominant. Comparing the three groups, the proportion of B cells, endothelial cells, Th and Treg cells decreased significantly in both the pulmonary and extra-pulmonary groups. In contrast, basophil, macrophage, monocyte and DC increased in the extra-pulmonary group and decreased in the pulmonary group. Although neutrophils also increased in the pulmonary group, the trend was not as pronounced as in the extra-pulmonary group. These changes in the percentage of cells clearly show the difference between the two pathogenic factors in acute lung injury.

### 3.3 Differential analysis focusing on each cell type demonstrates in detail the great variability of the two acute lung injury models

**Figure 3:**
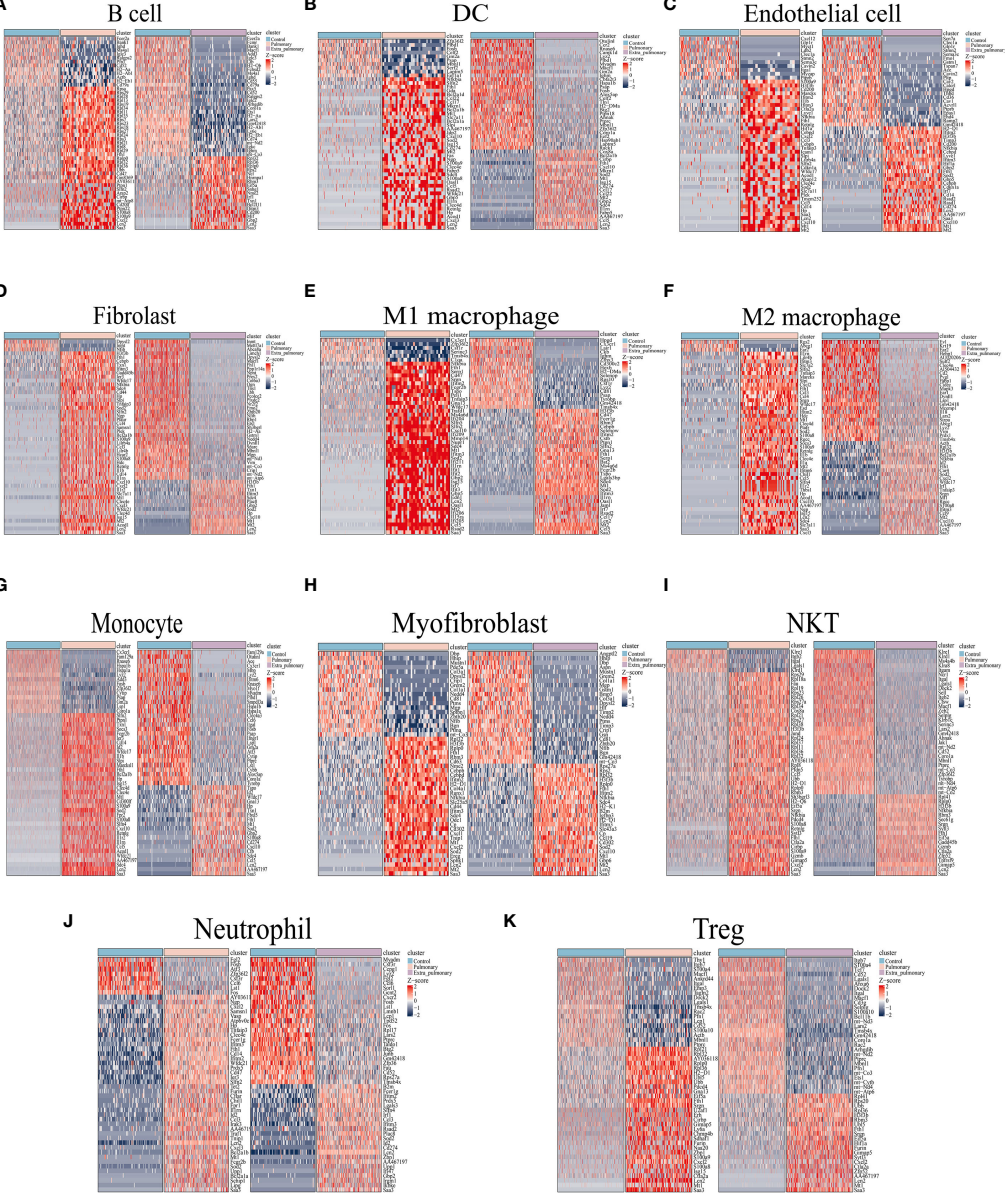
Differential genes in the pulmonary and extra-pulmonary acute lung injury relative to the control group for each cell type. Heatmaps representing the top 50 differentially expressed genes in **(A)** B cells, **(B)** DCs, **(C)** endothelial cells, **(D)** fibroblasts, **(E)** M1 macrophages, **(F)** M2 macrophages, **(G)** monocytes, **(H)** myofibroblasts, **(I)** NKT cells, **(J)** neutrophils, and **(K)** Treg cells. In the heatmap, light blue color represents the control group, light pink represents the pulmonary group, and light purple represents the extra-pulmonary group. The intensity of red color in the heatmap indicates higher expression levels, while the intensity of blue color indicates lower expression levels.

The separate differential analysis was conducted to pinpoint the variability of each cell type within the two groups, both inside and outside the lungs. Following p-value screening, no more than 500 differential genes (DEGs) were extracted from each cell population. Subsequently, the top 50 DEGs from each cell population were visualized using a heatmap (refer to **Figure 3**). Comparing the results of the single cell RNA-sequence and the bulk RNA-sequence, it can be found that while the top50 DEGs in the single-cell data of the pulmonary group had only 11 duplicates of the top50 genes of the bulk RNA-seq, such as Saa3, Lcn2,Cxcl1,Cxcl2, Ccl3, Mt2, Sod2, Tnfaip3, Ptafr, Il1rn, Cd14. Only 9 duplicates appeared in the extra-pulmonary group, such as Clec4a3, Plac8, Lair1, Cybb, Itgal, Irf7, Saa3,Zbp1, Lst1, Rsad2, Itgam. Observing and comparing DEGs in each population of cells in the pulmonary group, there are Ccl5, Cebpb, Clec4e, Cxcl10, Cxcl2, Fth1, Hp, Ifitm3, Il1rn, Lcn2, Mt1, Mt2, Nfkbia, Retnlg, S100a8, S100a9, Saa3, Sdc4, Slfn2, Sod2, and a number of other genes recurring in DEGs in multiple populations of cells, whereas the genes recurring in each population of cells in the lung outgroup were AA467197, Coro1a, Cxcl10, Fth1, Gbp2, H3f3b, Ifitm3, Lars2, Lcn2, Macf1, Mt1, Mt2, Ptprc, Saa3, Sdc4, Sod2. Contrasting DEGs in each population of the two groups individually, although there was a small overlap in DEGs in each population of cells, the percentage of different genes was much greater. In summary, acute lung injury caused by both intra-pulmonary and extra-pulmonary factors not only has vastly different gene expression at the overall level, but also in individual cell populations, and many of the differences are stabilized in multiple cell populations.

### 3.4 The different cellular interactions and signaling pathways in the different causes of acute lung injury

**Figure 4:**
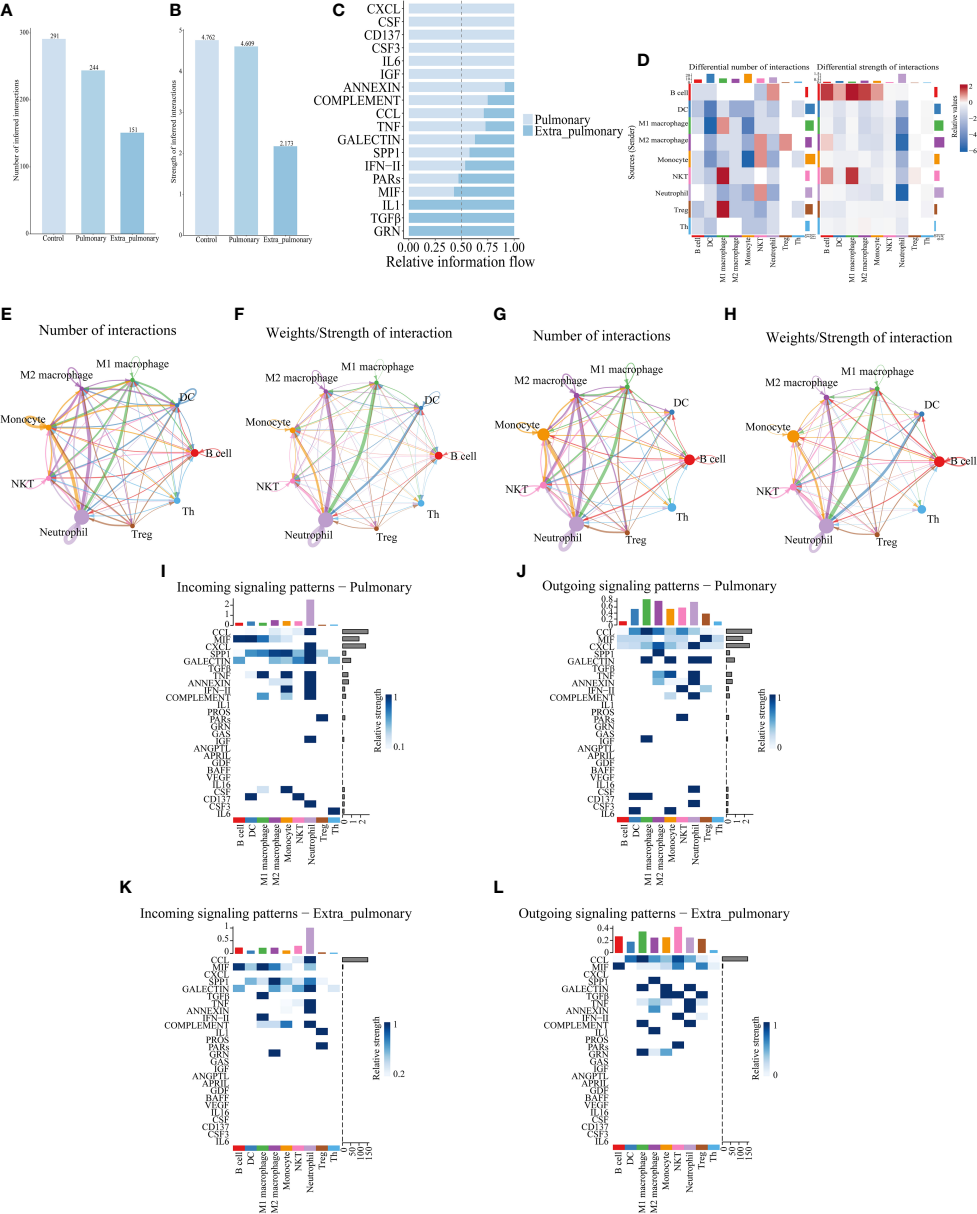
Cell communication analysis of single-cell data reveals differences between pulmonary and extrapulmonary acute lung injury. **(A, B)** Column graph of the total number or intensity of cellular communications in the three groups, with blue color representing the control, lung, and extra-lung groups in order from lightest to darkest; **(C)** Bar graphs of all signaling pathways interacting with cells within the pulmonary and extra-pulmonary groups, light blue represents the pulmonary group, dark blue represents the extra-pulmonary group, and the lengths of the bars represent relative expression; **(D)** Heat map of changes in the number and strength of cell interactions in the extrapulmonary group relative to the pulmonary group, with color blocks closer to red representing higher (stronger) in the extrapulmonary group and closer to blue representing higher (stronger) in the pulmonary group, and bars at the top of the heat map indicating how much/stronger the cell receives signals, and bars at the right indicating how much/fewer pairs of signals are emitted by the cell; **(E, F)** Network diagram of the number/intensity of cellular communication in the pulmonary group, with the size of the bubbles representing the corresponding number of cells, the color of the lines representing the cells that send out signals, the thickness of the lines representing the magnitude of the number/intensity, and the direction of the arrows representing the cells that receive signals; **(G, H)** Network diagram of the number/intensity of cellular communication in the extra-pulmonary group; **(I, J)** Heat map of the input/output signals of the cells in the pulmonary group, the closer the color block is to blue means the stronger the signal, the bar at the top of the heat map indicates the total intensity of the input/output signals of the cells, and the bar at the right indicates the total intensity of the signal’s input/output signals in the left and right cells; **(K, L)** Heat map of the input/output signals of the cells in the extra-pulmonary group.

Differential gene analysis through single-cell sequencing can solely capture variances between the two origins of ARDS from a singular viewpoint. Hence, we conducted cell communication analyses with the aim of unveiling distinctions in their intercellular signaling. Cellchat analysis revealed differences in the number and strength of cellular interactions between the control, pulmonary and extra-pulmonary group (**Figures 4A, B**). Relative information flow analysis showed that IL6, CSF3, CD137, and CSF were pathways specific to the pulmonary ALI compared to the control group. And CXCL, CSF, CD137, CSF3, IL6, IGF were pathways specific to the pulmonary ALI compared to the extra-pulmonary group. It can be seen that CD137, CSF3 and IL6 are signaling pathways specific to the pulmonary group but not to the remaining two groups. In contrast to the pulmonary group, IL1, TGFβ, and GRN are signaling pathways specific to the extra-pulmonary group (**Supplementary Figures S4A, B**, **Figure 4C**). In order to present more visually the difference in the strength of intercellular interactions between the pulmonary and extra-pulmonary groups, we used heatmaps and circle plots. Compared with the control group, the signal strength sent from M1 and M2 macrophages to neutrophils in the pulmonary group increased more significantly compared with the signal strength between other cells, whereas the signal strength sent from B cells to themselves, M1 macrophages and neutrophils in the extra-pulmonary group increased significantly. Signal strength of neutrophil autocrine secretion was significantly increased compared to the extra-pulmonary group. Compared with the pulmonary group, the signal strength sent from B cells to themselves and M1 macrophages in the extra-pulmonary group was increased, and the strength of the interaction between NKT cells and M1 macrophages also tended to increase. This shows that neutrophils, M1 and M2 macrophages play an important role in the pulmonary group, while B cells play an important role in cellular interactions in the extra-pulmonary group (**Figures 4D-H**, **Supplementary Figures S4C-F**). On the other hand, we further explored the incoming and outcoming signaling pathways involved in both the pulmonary and extra-pulmonary groups of cells. Through the presentation of heatmaps, it can be found that the main incoming and outcoming signaling pathways in the control group were CCL and MIF, and the main incoming and outcoming signaling pathways in the pulmonary group were CCL, MIF and CXCL, while the main ones in the extra-pulmonary ALI were CCL and MIF. In the pulmonary ALI mice, neutrophils acted on themselves mainly through CXCL and CSF3. While in extra-pulmonary ALI mice, B cells mainly acted on M1 macrophages through the MIF signaling pathway, while NKT cells mainly acted on M1 macrophages through the TGFβ signaling pathway function (**Figures 4I-L**, **Supplementary Figures S4G, H**). Taken together, our analyses showed that cellular interactions between the pulmonary ALI, extra-pulmonary ALI, and control mice differed significantly in terms of cellular involvement in terms of types, strength and signaling pathways.

### 3.5 Neutrophil autocrine secretion plays a crucial role in acute lung injury due to intrapulmonary factors

**Figure 5:**
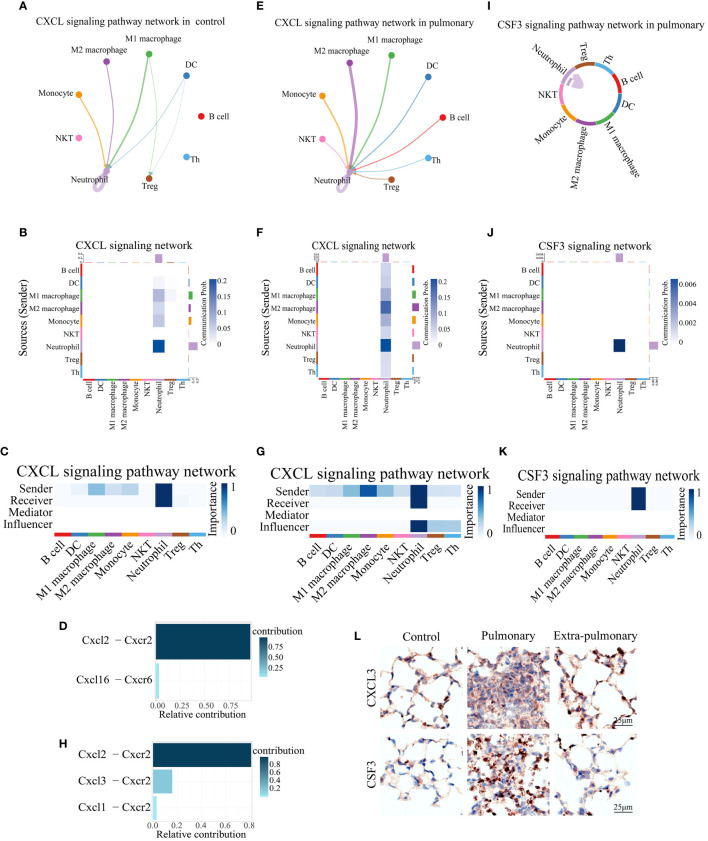
Both of CXCL and CSF3 pathways showed much higher strength in neutrophil autocrine in pulmonary acute lung injury. **(A, E)** Network diagram of the communication strength of CXCL signals between control and pulmonary groups in each population of cells, with the color of the line representing the cell from which the signal is emitted, the thickness representing the strength, and the arrow pointing to the receiver of the signal; **(B, F)** Heatmap of the communication strength of the CXCL signal between the control and pulmonary groups in each population of cells, the closer the color block is to the blue the stronger the signal, the bar at the top of the heatmap indicates the total strength of the CXCL signal received by each population of cells, and the bar on the right indicates the total strength of the CXCL signal emitted by each population of cells; **(C, G)** Heatmap of the roles played by each population of cells in the CXCL signaling pathway and their share of the CXCL signaling pathway in the control and pulmonary groups, with the closer the color block is to blue indicating a greater share of the CXCL signaling pathway; **(D, H)** Bar graph of the contribution of ligand receptors functioning in the CXCL signaling pathway in the control and the pulmonary groups, with darker colors, more upward positions, and longer bars indicating greater contributions; **(I)** Chordal diagram of cellular communication in the CSF3 signaling pathway in the pulmonary group, with colors representing cells that send signals and arrows pointing to cells that receive them; **(J)** Heatmap of cellular communication in the CSF3 signaling pathway in the pulmonary group; **(K)** Heatmap of pulmonary group of cells that play a role in the CSF3 signaling pathway; **(L)** Immunohistochemical staining of CXCL3 and CSF3 pathways in control, intrapulmonary and extrapulmonary groups.

We then analyzed the two dominant pathways in the pulmonary group in more detail. It can be seen that CXCL is predominantly emitted by monocytes, macrophages, DCs and neutrophils in the control group and received by neutrophils and Treg cells (**Figures 5A-C**). Based on the thickness of the lines and the shade of the heat map color it can be seen that autocrine secretion emitted by neutrophils to neutrophil receptivity is the strongest signal in all pairs of interacting cells (**Figures 5A, B**). The Cxcl2-Cxcr2 ligand receptor pair plays a major role in its signaling pathway, with Cxcl6-Cxcr6 playing a minor role (**Figure 5D**). When CXCL was grouped in the lung, the senders of the signal were neutrophils, NKT cells, monocytes, macrophages, DCs, B cells, Th cells, and Treg cells, and the receivers were neutrophils as the only ones (**Figures 5E-G**). Compared to the control group, Treg shifted from being receivers of signals to senders of signals, while Th cells, B cells and NKT cells began to play a role in the pathway. Although the autocrine effect of neutrophils continued to be the strongest in terms of signal intensity, the secretory effect of M2 macrophages on neutrophils was markedly enhanced compared with controls, which is also noteworthy (**Figures 5E, F**). At the same time the ligand receptors within the pathway changed significantly, the Cxcl2-Cxcr2 signaling did not change significantly, but the Cxcl6-Cxcr6 signaling disappeared, and in its place it functioned as the two ligand receptor pairs, Cxcl3-Cxcr2 and Cxcl1-Cxcr2 (**Figure 5H**). Since the Cxcl signaling pathway was not present in the extra-pulmonary group, graphs related to this pathway in the extra-pulmonary ALIwere not plotted.

Similarly, for the other dominant pathway, CSF3, its pictures in the control and extra-pulmonary ALI were not plotted as it was not present in the control and extra-pulmonary ALI. Viewing the form of action of CSF3 in the pulmonary group it can be seen that unlike the other pathways, this pathway only has lines pointing to neutrophils (**Figure 5I**) and the heatmap also only has a clear color block at neutrophils (**Figures 5J, K**). Suggesting that it is a specific neutrophil autocrine pathway of action. Although both pathways, CXCL and CSF3, are dominant signaling in the pulmonary ALI, their modes of action in the pulmonary ALI mice are still quite different.

Overall, both of CXCL3 and CSF3 showed much higher strength in pulmonary group than other group, which can be seen in violin maps of their gene expression and immunohistochemical staining of lung tissue (**Figure 5L**, **Supplementary Figures S5A-C**). This may provide a feasible idea for the treatment of ALI/ARDS caused by intra-pulmonary factors.

### 3.6 The MIF and TGFβ signaling pathways indicate great significance in the extra-pulmonary acute lung injury

**Figure 6:**
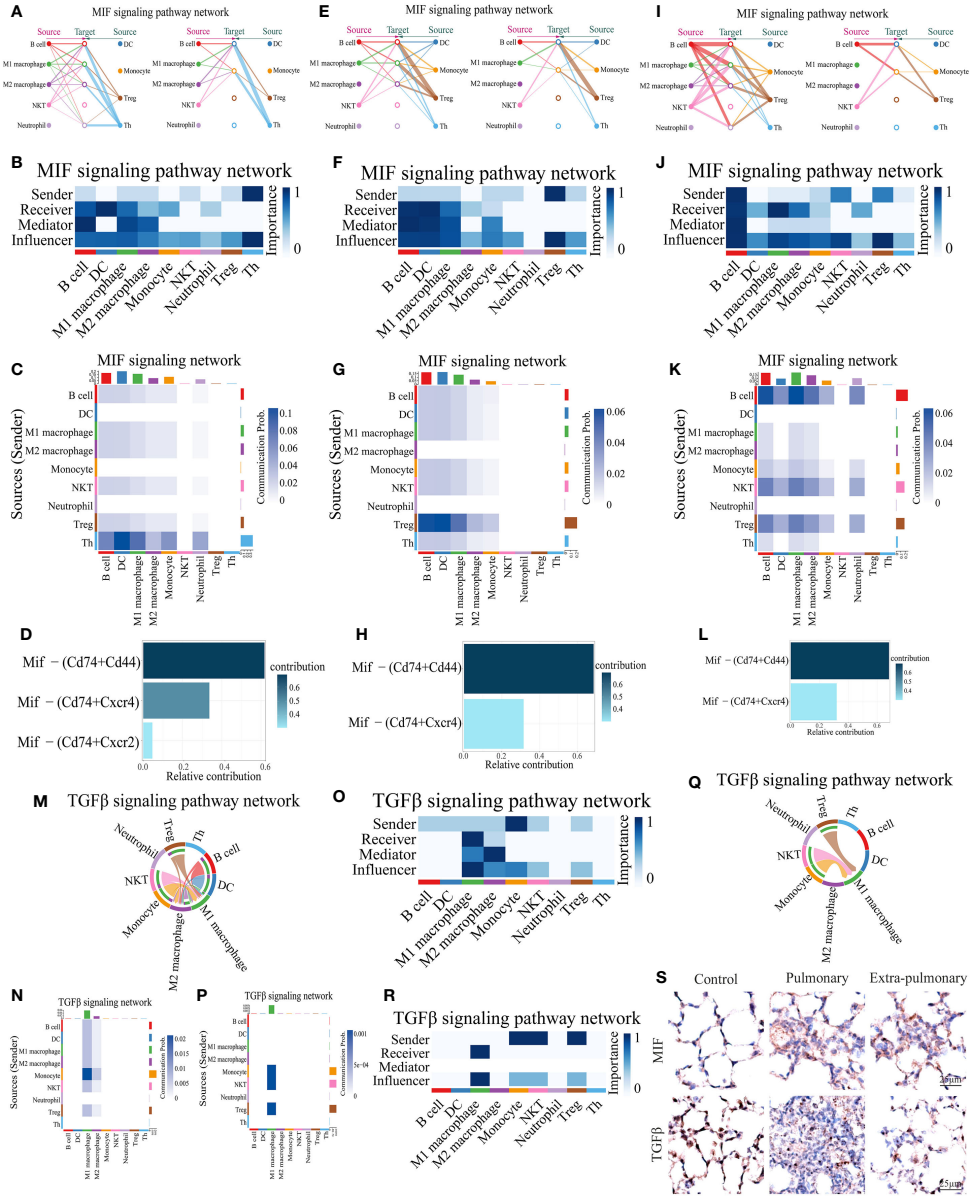
Analysis of predominant immune cells and their signaling pathways in extra-pulmonary acute lung injury. **(A, E, I)** Diagram of the cellular communication network of the MIF signaling pathway in the control, pulmonary, and extra-pulmonary groups, with solid circles representing the senders of signals and hollow circles representing the receivers of signal; **(B, F, J)** Heatmap of control, pulmonary and extra-pulmonary cells that take roles in the MIF signaling pathway; **(C, G, K)** Heatmap of the MIF signaling pathway in cellular communication in the control, pulmonary and extra-pulmonary groups; **(D, H, L)** Histogram of MIF signaling pathway ligand receptor contribution to cellular communication in control, pulmonary and extra-pulmonary groups; **(M, Q)** TGFβ cell communication network map in control and extra-pulmonary groups; **(N, P)** Heatmap of the TGFβ signaling pathway in cellular communication in the control and extra-pulmonary groups; **(O, R)** Heatmap of control and extra-pulmonary cells that take roles in the MIF signaling pathway; **(S)** Immunohistochemical staining of MIF and TGFβ pathways in control, intrapulmonary and extrapulmonary groups.

ARDS induced by extra-pulmonary factors demonstrates notable distinctions in comparison to that triggered by pulmonary factors. In the former, pathways related to CXCL and CSF3 are less prominent, while pathways linked to MIF and TGFβ signaling are more distinctive. From the performance of MIF in the control group, it can be seen that its main emitting cells are Th cells, all of which have some influence on the pathway to varying degrees, but DC is its main recipient (**Figures 6A-C**). For this pathway Mif-(Cd74+Cd44) is its predominant co-receptor pair, while Mif-(Cd74+Cxcr4) and Mif-(Cd74+Cxcr2) also play a role in the pathway (**Figure 6D**). When the MIF pathway was present in the pulmonary ALI group, a shift of the main signal emitter to Treg cells could be noticed, the intensity of the DC, the receiver of the signal, did not change, but the signal received by the B cells was significantly enhanced, and the neutrophils no longer received the signal from MIF (**Figures 6E-G**).Mif-(Cd74+Cd44) and Mif-(Cd74+ Cxcr4) signal intensities in the pathway did not change significantly, but Mif-(Cd74+Cxcr2) ceased to play a role (**Figure 6H**). Observing the MIF signaling pathway in the extra-pulmonary group, it can be seen that the main sender of this signaling pathway changed to B cells, while NKT and Treg cells also played a non-negligible role in it (**Figures 6I-K**). The receivers of this signaling pathway in the extra-pulmonary group were mainly B cells and M1 macrophages (**Figures 6I-K**). The co-receptors that play a role in this are the same as in the pulmonary group (**Figure 6L**).

The TGFβ signaling pathway was not expressed in the pulmonary group, so only the control and extra-pulmonary groups were plotted. Viewing the TGFβ signaling pathway in the control group, it can be seen that the main signal recipients of the pathway are macrophages, with M1 macrophages playing a greater role, and all cells involved in the analysis except neutrophils and Th cells emitted the signal, with the strongest signal coming from monocytes (**Figures 6-O**). The co-receptor pair for this pathway is borne by TGFβ1- (TGFβr1+TGFβr2) (**Supplementary Figure S6A**). In contrast, when TGFβ appeared in the extra-pulmonary group, both the sender and receiver of the signal changed significantly, and the heatmap and chord diagrams showed that only monocytes, NKT cells, and Treg cells emitted the signal, and there was no significant difference in the signal intensity of the three, whereas the M2 macrophage no longer received the signal, and only the M1 macrophage was left as the receiver of the signal (**Figures 6P-R**). However, the co-receptor of the lung outer group did not change and remained TGFβ1 - (TGFβr1+TGFβr2) (**Supplementary Figure S6B**).

Collectively, both the MIF and TGFβ pathways exhibited significant alterations in the extra-pulmonary ALI mice. Particularly, the MIF signaling pathway displayed notable differences across all control, pulmonary, and extra-pulmonary ALI groups, although its overall expression did not show a significant increase post-injury. In contrast, the TGFβ signaling pathway exhibited a high specificity for M1 macrophages in the extra-pulmonary ALI mice, with an overall decreased volume. Violin plots illustrating gene expression of the signaling factors involved in these pathways correspond to the aforementioned findings. These trends are also evident in the immunohistochemical images of the respective lung tissues (**Figure 6S**, **Supplementary Figures S5A-C**).

### 3.7 The scRNA-seq analysis of the human sample confirmed the critical role of the CXCL and MIF pathways in ARDS

**Figure 7:**
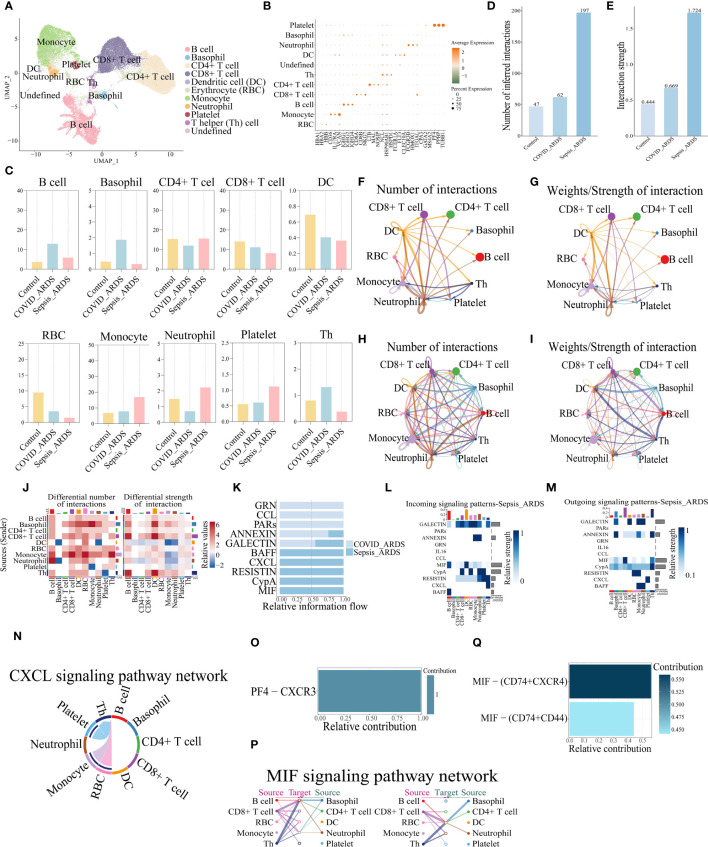
MIF and CXCL signaling pathways were also identified in the analysis of human peripheral blood single-cell sequencing data. **(A)** UMAP dimensionality reduction map after cell clustering; **(B)** Bubble plot of cellmarker gene expression; **(C)** Bar graph of percentage of cells per population in control, COVID-19-associated and sepsis-related ARDS groups; **(D, E)** Bar graphs of the total number and intensity of cellular communications in the three groups; **(F-I)** Network plot of the number and intensity of cell interactions in the COVID-19-associated and sepsis-related ARDS groups; **(J)** Changes in the number and intensity of cellular interactions in the sepsis-related ARDS group compared to the COVID-19-associated ARDS group; **(K)** Bar graph of signaling communication between COVID-19-associated and sepsis-related ARDS group cells; **(L, M)** Heatmap of signaling communication between COVID-19-associated and sepsis-related ARDS group cells; **(N)** Chordogram of CXCL cell communication in sepsis-related ARDS group; **(O, Q)** Histogram of the contribution of the pair of ligand receptors for CXCL and MIF cell communication in the sepsis-related ARDS group; **(P)** Communication network diagram of MIF cells in sepsis-related ARDS group.

To demonstrate whether the results of mice with ALI analysis are also reproducible in humans, the peripheral blood sequencing results of patients with sepsis complicating ARDS (GSE151263) and patients with COVID-19 complicating ARDS (GSE150728) were downloaded from the Gene Expression Omnibus (GEO) database and merged for Analysis. B cells, Neutrophil, Platelet, CD8+ T cells, CD4+ T cells, Basophil, Erythrocyte (RBC), Dendritic cell (DC), Monocyte, T helper (Th) cell totaling 10 types of cells (**Figures 7A, B**).The changes in their number of cells per population were counted and plotted (**Figure 7C**, **Supplementary Figure S7**), which showed a similar trend of decreasing numbers in both COVID-related and sepsis-related ARDS patients, which was slightly different from the analysis of mice. Analyzing their cellular communication, we found that the number and intensity of cellular communication increased in both COVID-19-associated ARDS and sepsis-related ARDS patients (**Figures 7D-I**), with the most pronounced elevation in sepsis-related ARDS patients, which was three times higher than that of COVID-19-associated ARDS patients. Viewing the cells that play a major role in cellular communication in its two groups relative to the control group we can see that in the COVID-19- associated ARDS group the main emitters of signals were monocytes, DC and CD8+ T cells, and the main receivers were monocytes and neutrophils (**Supplementary Figures S8A-C**). In contrast, the main emitters of signals in the sepsis-related ARDS group were basophils, CD8+ T cells and Th cells, and the main receivers were B cells and DC cells (**Supplementary Figure S8D**). Comparing the two groups, the cell pairs with stronger interactions in the pulmonary group of COVID-ARDS were DC cells acting on monocytes or neutrophils, and autocrine secretion by monocytes. In contrast, the cell pairs that interacted more strongly in the extra-pulmonary of sepsis-related ARDS group were basophils, CD8+ T cells and Th cells acting on DC cells, and CD8+ T cells and Th cells acting on B cells (**Figures 7F-J**). Comparing the signaling pathways in these two groups, it can be seen that all three pathways, GRN,CCL, and PARs, are specifically expressed in the COVID-19-associated ARDS, while BAFF, CXC, LRESITIN, CypA, and MIF are specifically expressed in the sepsis-related ARDS (**Figure 7K**, **Supplementary Figures S8E-J**). It is noteworthy that in mice CXCL was the pathway expressed in the pulmonary ALI, whereas MIF was expressed in all of control, pulmonary and extra-pulmonary ALI, except in the extra-pulmonary ALI where it was at a higher state of expression, whereas in human samples both CXCL and MIF were sepsis-related ARDS group-specific. About the CXCL pathway signals in the sepsis-related ARDS, we see that it signals in the direction of platelets, RBCs and monocytes acting on Th cells, with the strongest signals emitted by platelets (**Figures 7L-N**, **Supplementary Figures S8K, L**), and the ligand receptor that plays a role within its pathway is PF4-CXCR3 (**Figure 7O**). Analyzing the MIF signaling pathway, we see that MIF is mainly emitted by CD8+ T cells and Th cells and received by B cells and DC cells (**Figures 7L, M, P**, **Supplementary Figures S8M, N**). The ligand acting within its pathway is MIF and the receptors are CD74,CD44 and CXCR4, the ligand-receptor pair that plays a major role is MIF-(CD74+CXCR4) (**Figure 7Q**), whereas the mouse data analyzed shows that MIF-(CD74+CD44) plays a major role in the extra-pulmonary ALI mice.

## 4 Discussions

Based on our research, both pulmonary and extra-pulmonary acute lung injury models display complex changes in cell populations, with consistently lower levels of Th cells, Treg cells, and B cells compared to the control group. This consistent finding potentially indicates that the systemic inflammatory reaction in ARDS may induce an immune suppression response (30). However, extra-pulmonary ALI is characterized by a significant increase in monocytes, macrophages, and epithelial cells. In contrast, pulmonary ALI shows a notable relative increase in neutrophils, which appears to be closely associated with the lack of a prominent rise in macrophages (31). Overall, the numerical alterations in these cell populations associated with inflammation align with the increased levels of inflammatory markers in the bloodstream observed in sepsis-related ARDS patients as compared to those with pneumonia-induced ARDS in clinical settings (7–9, 32–35).

Differential analysis of the above single-cell data showed high frequency occurrence of Clec4e, Retnlg and S100a9 as genes unique to the top50 DEGs in the pulmonary group, whereas Coro1a and Lars2, were genes that were highly frequent and unique to the top50 DEGs in the extra-pulmonary ALI. These five genes may hold promise as potential targets for the diagnosis or treatment of ARDS caused by different factors. C-type lectin receptor 4e (Clec4e) recognizes signaling factors released by necrotic cells to mediate the inflammatory response. It has been shown that Clec4e in macrophages can be stimulated by elevated lipopolysaccharide, promoting macrophage proliferation and activation of inflammatory responses (36). While in Inflammatory epidermolysis bullosa acquisita although knockdown of Clec4e does not prevent disease progression, it can be relied on the transcriptional expression of Clec4e to determine whether neutrophils in the bloodstream are activated and enter the dermis (37), and in the model of myocardial ischemia reperfusion an investigator found that there is a certain relationship between the amount of expression of Clec4e and the area of infiltration of neutrophils, and that the treatment targeting Clec4e could be effective in improving the inflammation of myocardium after myocardial ischemia reperfusion injury (38). Previous studies have illustrated that Clec4e is closely associated with macrophages and neutrophils, and that neutrophils in different disease models have different properties. Comparative analysis of the differences between our three groups of mouse models revealed that M1 and M2 macrophages as well as neutrophils of mice in the pulmonary group showed high expression of Clec4e, and that this gene was not found in all DEGs of the three types of cells of mice in the extra-pulmonary group. Perhaps Clec4e expression in macrophages and neutrophils can be used as an indicator to identify the different factors contributing to ARDS. Retnlg has been shown to be associated with neutrophil migration (39, 40), whereas in our study, Retnlg present in top50 DEGs of multiple cell clusters in the pulmonary ALI, was not found in the DEGs of neutrophils. S100 Calcium Binding Protein A9 (S100a9) usually present as a heterodimer *in vivo*, mainly released by immune cells, such as neutrophils and macrophages (41), and is an early-warning protein for inflammation, initiated by Toll-like Receptor 4 (TLR4) in response to LPS stimulation (42). Blockade of S100a9 in a mouse model of LPS-induced ALI effectively reduced lung injury by inhibiting the Nlrp3 pathway (43), and S100a9 was identified as having potential diagnostic and prognostic value in a study on ARDS proteomics (44). However, S100a9 was not only present in the ARDS mouse model in the pulmonary ALI. Although S100a9 was not detected in the top50 DEGs of our extra-pulmonary ALI model, it was present in all DEGs of the extrapulmonary ALI. Inhibition of S100a9 has been found to be effective in decreasing lung neutrophil infiltration and lung injury in mice with sepsis-related ARDS in a study of sepsis (45), and the same finding has been found in a model of myocardial infarction (46). Perhaps S100a9 could be a generalized target for the treatment of ARDS caused by both pulmonary and extra-pulmonary causes. Coro1a belongs to the actin-binding protein family (47) and is mainly expressed in monocytes, NK cells, T cells, B cells, DCs and macrophages (48, 49). Coro1a regulates actin cytoskeleton-dependent processes such as cytokinesis, cell polarization, migration, and phagocytosis, and its absence effectively attenuates neutrophil infiltration during innate immunity (49). However, as far as the current studies are concerned, only in chronic obstructive pulmonary emphysema (COPD) and chronic pulmonary fibrosis has there been a focus on the great role of Coro1a in immune cells, and its role has not yet been investigated in the disease of ARDS. There are fewer studies on the relationship between Lars2 and the immune response, but it is noteworthy that some studies have proposed that the expression of TGFβ1 is elevated in Las2-positive B cells, which in turn is a driver of the differentiation of CD4+ T cells into Treg cells (50). This is in line with the results of the present study, in which we found that the expression of Las2 in B cells was decreased in the extrapulmonary lung injury in the analysis of the present mouse data, while the clustering analysis of single cells showed that the relative number of Treg cells in the extrapulmonary ALI was also downregulated compared with the control group. However, the role of Las2 in ARDS remains to be investigated. We also investigated the immune cell populations to identify the signaling molecules or pathways that are most differentially and critically important for intracellular communication.

The key roles played by CXCL,CSF3,MIF and TGFβ in both ARDS can also be seen by communication analysis of single cell data and staining of lung tissue. Our findings suggest that CXCLs play a vital role in attracting and activating different types of immune cells, including neutrophils and T cells, which leads to the initiation of an inflammatory response. This mechanism is involved in the development of various inflammatory diseases, accompanied by tissue damage (51). For example, previous research has shown that the COVID-19-associated ARDS exhibited higher levels of plasma CCL5, CXCL2, and CXCL10 compared to the non-COVID-19 ARDS patients. Additionally, the concentrations of CXCL1 and CXCL10 in the epithelial lining fluid (ELF) were also elevated in the COVID-19-associated ARDS. CXCL10 shows potential as a biomarker for predicting the duration of mechanical ventilation in patients with COVID-19-associated ARDS (52). COVID-19-associated ARDS exhibits distinct characteristics that suggest an unusual immune response. However, in our study, we found that the mouse model of ALI due to intra-pulmonary factors exhibited a significant increase in the expression of CXCL1 and CXCL3, and CXCR2 expression was increased, which are consistent with published experimental results (53–57). The examination of peripheral blood samples from individuals with COVID-19-associated ARDS did not show activation of the CXCL pathway. This absence of activation could be attributed to the viral nature of the samples used in this analysis. Conversely, in blood samples from patients with sepsis, we observed activation of the CXCL pathway along with a notable rise in the ligand PF4. This discovery, previously undocumented, has enabled us to identify specific key factors through which ischemia contributes to the advancement of sepsis-related ARDS. However, whether based on previous studies or the present study, the role played by CXCL1 in the CXCL pathway can be affirmed. Considering the functional properties of the CXCL1, we observed that palmatine has the ability to reduce the expression of CXCL1 and CXCL2. This reduction in expression leads to decreased infiltration of inflammatory cells and alleviation of lung injury induced by lipopolysaccharide (58). A potential new therapeutic approach that could be considered is focusing on the CXCL axis.

Granulocyte-macrophage colony-stimulating factor (CSF) (also known as CSF2) and granulocyte-CSF (also known as CSF3, G-CSF) are important survival and proliferation factors for neutrophils and macrophages (59), and deficiency of CSF3 leads to neutropenia (60, 61). And mortality in ARDS patients was positively correlated with the amount of CSF3 signaling factor in their lung tissues (62). However, stimulation of neutrophils by CSF3 in a rat model aggravated ventilator-induced lung injury, as evidenced by increased lung neutrophil and interleukin-6 expression, increased histologic alveolar edema, and decreased lung compliance (63). In patients with ARDS, the level of CSF3 expression in the lungs correlates with the severity of pulmonary neutrophilia, and elevated neutrophils correlate with a poorer prognosis (61). It has been proposed that CSF can be used as an early warning marker of whether COVID-19 causes myocardial injury (64), or to treat COVID-19 by interfering with the CSF pathway (65), but neither CSF nor CSF3 was found in the results of our analysis of human single-cell data. In contrast, in our study about ALI mice, we found that CSF3 was the highest autocrine signal for neutrophils in the pulmonary ALI and that neutrophils were the only cells in which the CSF3 pathway was present. And it has been shown that inhibition of the CSF3 pathway in ARDS disease inhibits neutrophil accumulation in the lungs (62). Based on the above findings, perhaps we have found a way to effectively reduce neutrophil production and aggregation during the treatment of ARDS due to intrapulmonary factors, and attenuate neutrophil accumulation in the alveoli through inhibition of the CSF3 pathway, in order to effectively improve the prognosis of the disease.

Macrophage Migration Inhibitory Factor(MIF) is a potential secreted molecule involved in most immune cell communication in extra-pulmonary ALI. MIF is an inflammatory cytokine with enzymatic activity and can be secreted by a variety of cell types, such as monocytes, macrophages, DC, B cells, neutrophils, Treg, and NKT (66). Due to the absence of a signal peptide, MIF is stored within intracellular vesicles when the cells are at rest. However, upon cellular stimulation, MIF is released into the extracellular space through exosomes. In a study published in 2021, the authors highlighted the significant role of the MIF signaling pathway in sepsis. Their conclusions also underscored the substantial difference in the expression of the MIF-CXCR2 ligand-receptor pair between the control and disease groups (67, 68). Our study reaffirms that both in mice and humans MIF exerts its effects on downstream receptors CD74, CD44, CXCR2 and CXCR4 via autocrine or paracrine signaling pathways (66). MIF has an important function as a crucial regulatory element in the host’s innate and adaptive immune responses, playing a role in modulating TLR4 expression in mouse monocytes (69). Abnormal expression of MIF has been linked to a range of diseases, including sepsis, myocardial infarction, acute kidney injury, organ fibrosis, malignant tumors, rheumatoid arthritis, systemic lupus erythematosus, and inflammatory bowel disease (66). The levels of MIF in the bloodstream are increased in patients with sepsis and are linked to negative clinical outcomes (70). By using anti-MIF antibodies to neutralize MIF, it has been demonstrated that TNF-α production and the build-up of neutrophils can be reduced, resulting in the alleviation of symptoms and improved prognosis for sepsis-related ARDS patients, ultimately reducing the likelihood of mortality. It has been demonstrated that MIF serves as a crucial biomarker in acute lung injury. Elevated MIF levels were significantly observed in sepsis and sepsis-related ARDS in humans, as well as in LPS-induced mouse models of ALI (71). Similarly, heightened MIF levels were noted in rat models induced by intraperitoneal injection of LPS, where MIF antibodies notably mitigated migration of neutrophils from blood vessel to the pulmonary alveolus (72). Our analysis suggests that the communication score of MIF is significantly elevated, with neutrophils being the primary recipients of MIF in cases of extra-pulmonary ALI. This reaffirms the critical involvement of the MIF signaling pathway for neutrophils and underscores the pivotal role of neutrophils in ARDS induced by extra-pulmonary factors. This stands in contrast to the prior assumption that neutrophils primarily contribute to ARDS triggered by pulmonary factors.

Transforming growth factor b (TGFβ) is a cytokine belonging to the transforming growth factor family, which has a key function in regulating inflammatory processes and also plays a crucial role in T cell regulation and differentiation (73, 74). It has been shown that Treg is the primary source of TGFβ in the process of immune regulation in the body (75), and TGFβ plays an important role in maintaining the stability and function of Treg (74). In the present study, we found that the TGFβ signaling pathway has an extremely important role across multiple cells in the normal organism, but during disease progression of ARDS due to extra-pulmonary factors, the major changes in the TGFβ signaling pathway were focused on signaling from NKT and Treg cells to M1 macrophages. In terms of published studies, although the role of TGFβ in sepsis-related ARDS has been demonstrated, most investigators have focused on the function of M1 macrophages as senders rather than receivers of TGFβ signaling, whereas, according to our findings, blocking the reception of TGFβ signaling by M1 macrophages may be important for the treatment of extrapulmonary factors that contribute to ARDS.

In summary, we have identified five additional differentially expressed genes—Clec4e, Retnlg, S100a9, Coro1a, and Lars2—in control, intrapulmonary, and extrapulmonary LPS intervention mouse models. Furthermore, four signaling pathways—CXCL, CSF3, MIF, and TGFβ—have been pinpointed as significant contributors to the heterogeneity of ARDS stemming from these two causes. Various methodologies are available to induce ARDS in mouse models through extrapulmonary factors, such as oleic acid tail vein injection, cecal ligation and puncture (CLP), and intraperitoneal LPS administration (76–80). Similarly, simulating ARDS due to intrapulmonary factors involves intratracheal injection of LPS and mechanical ventilation-induced lung injury modeling (81, 82). In our study, we focused solely on the LPS-induced approach for modeling and data analysis to maintain model stability, generalizability, and data reproducibility, despite some limitations. Future studies should explore the consistency of data obtained from alternative ARDS modeling methods for the two factors.

## 5 Conclusions

**Figure 8:**
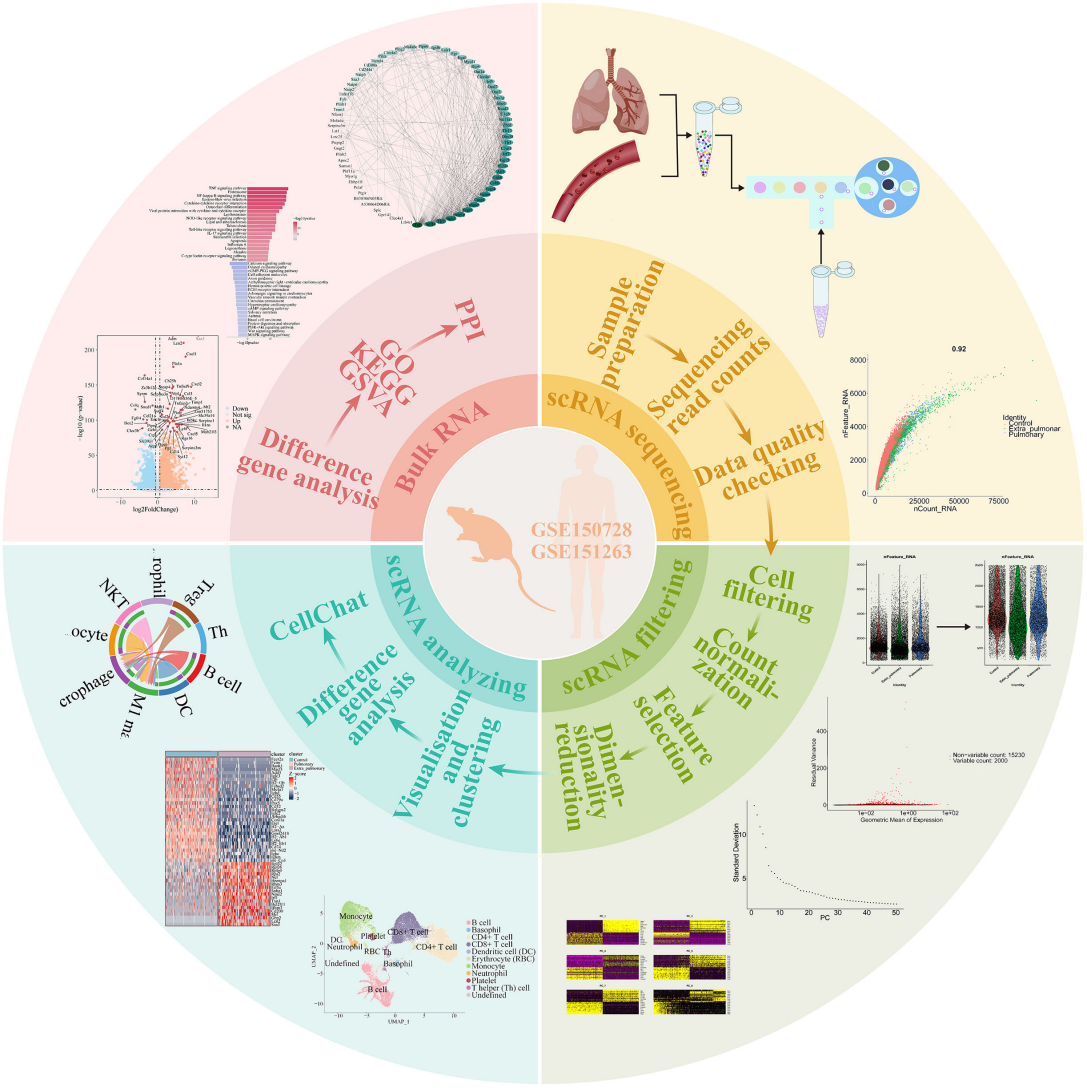
Graphical abstract. The analysis of ALI from both tissue and single-cell perspectives revealed variations in ALI caused by different factors. It was observed that the cellular composition of distinct ALI subtypes varies. Furthermore, significant differences were identified in gene expression and pathway activation among different subtypes of ALI. Comparison of the strength of cellular communication showed that ALI caused by pulmonary factors was characterized by a higher intensity of neutrophil autocrine secretion, whereas ALI caused by extra-pulmonary factors showed a stronger effect of B cells and NKT cells on M1 macrophages. Based on the comparison of the intensity of cellular communication, CXCL and CSF3 are more valuable in ALI caused by pulmonary factors, and MIF and TGFβ are more valuable in ALI caused by extra-pulmonary factors. The single-cell analysis of human peripheral blood confirmed the findings mentioned above. However, it is important to highlight that the MIF and CXCL pathways were also observed in the sepsis-related ARDS patients.

The analysis of bulk and single-cell transcriptomics has shown significant heterogeneity in the transcriptional profiles and cellular interactions among the various causes of acute lung injury in mice and human (**Figure 8**). These findings have potential implications in translating biological subphenotypes into clinical practice for patients with acute respiratory distress syndrome.

## Data availability statement

The data presented in the study are deposited in the GEO-NCBI repository, accession numbers GSE263867 and GSE264032.

## Ethics statement

The animal experiments were approved by the Ethics Committee of the Second Affiliated Hospital, Zhejiang University School of Medicine (No.2022050). The studies were conducted in accordance with the local legislation and institutional requirements. Written informed consent was obtained from the owners for the participation of their animals in this study.

## Author contributions

Z-YK: Data curation, Methodology, Validation, Visualization, Writing – original draft. Q-YH: Data curation, Methodology, Validation, Visualization, Writing – original draft. N-XZ: Methodology, Validation, Writing – review & editing. N-XX: Methodology, Validation, Writing – review & editing. Q-CZ: Methodology, Validation, Writing – review & editing. JZ: Methodology, Validation, Writing – review & editing. WC: Supervision, Writing – review & editing. Z-CZ: Conceptualization, Funding acquisition, Methodology, Writing – review & editing, Validation. B-PT: Conceptualization, Funding acquisition, Investigation, Methodology, Project administration, Supervision, Writing – review & editing, Validation, Visualization.

## Supplementary material

Supplementary Figure S1 Quality control and filtering of mouse lung tissue single-cell sequencing data.

Supplementary Figure S2 Quality check of mouse model and high throughput data.

Supplementary Figure S3 Number of mouse lung tissue cells in the control, pulmonary, and extra-pulmonary groups and their percentage in the groups.

Supplementary Figure S4 The results of cellular communication in the control, pulmonary and extra-pulmonary groups showed a huge difference.

Supplementary Figure S5 Gene expression of Mif, Cxcl1, Cxcl2, Cxcl3 and Csf3 in each population of cells.

Supplementary Figure S6 Histogram of ligand receptor contributions to the TGFβ signaling pathway in the control and extra-pulmonary groups.

Supplementary Figure S7 Number of human peripheral blood cell in the control, COVID-19-associated ARDS and sepsis-related ARDS groups and their percentage in the group.

Supplementary Figure S8 Cellular communication results of control, COVID-19-associated ARDS and sepsis-related ARDS showed obvious differences.
