# Supplementary material for: Heterogeneity of immune cells and their communications unveiled by transcriptome profiling in acute inflammatory lung injury
Source: Front Immunol. 2024 Apr 30;15:1382449. doi: 10.3389/fimmu.2024.1382449 (PMC11092984; doi:10.3389/fimmu.2024.1382449)
Supplement: Supplementary Figure S1 — Quality control and filtering of mouse lung tissue single-cell sequencing data. [file Presentation_1.pdf]

frontiers

Supplementary Material

Supplementary Figures

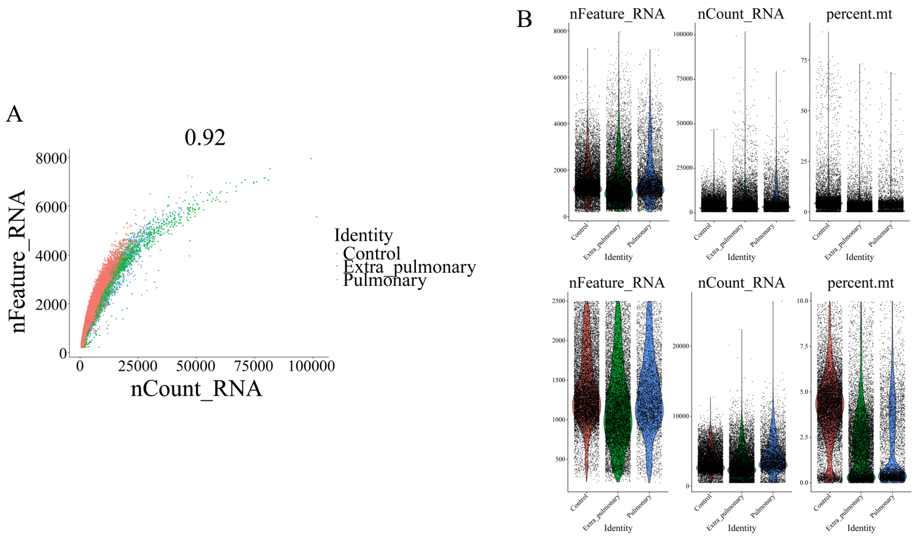

SUPPLEMENTARY FIGURE S1. Quality control and filtering of mouse lung tissue single-cell sequencing data. (A) Scatterplot of the correlation between the number of transcripts and the number of genes, with numbers closer to 1 indicating better quality data. (B) Violin diagrams before and after removal of mitochondrial genes.

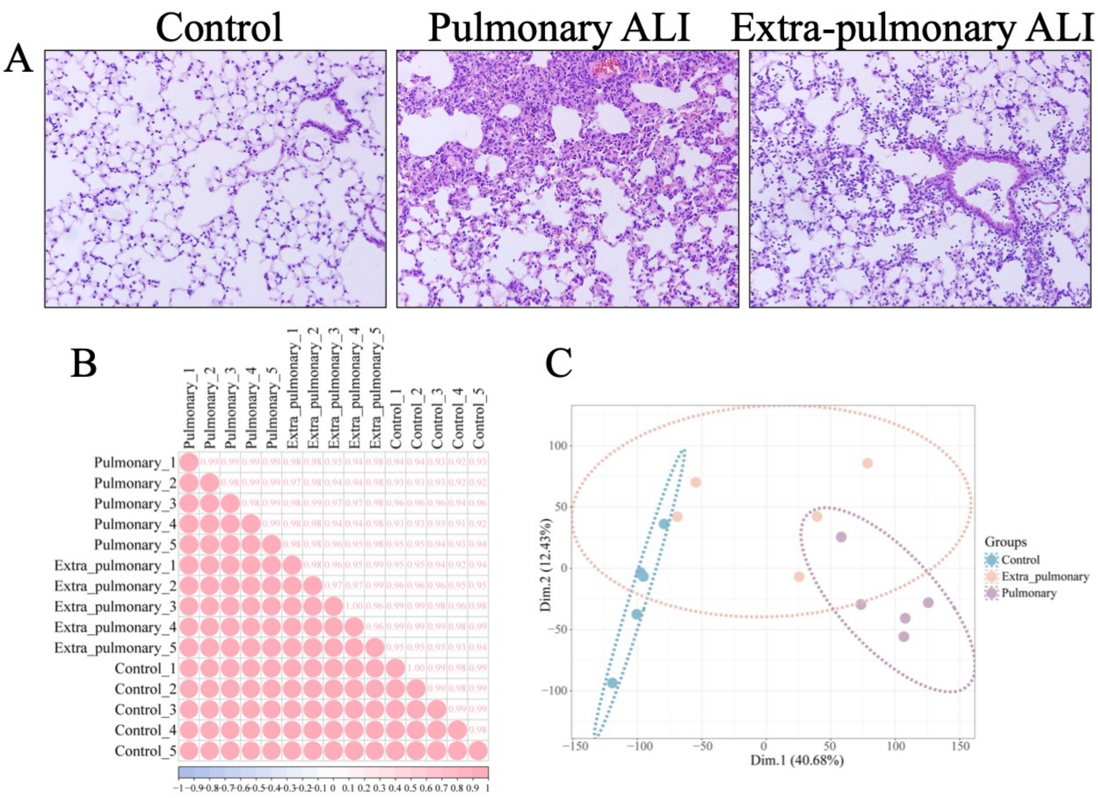

SUPPLEMENTARY FIGURE S2. Quality check of mouse model and high throughput data. (A) HE staining of paraffin sections of lung tissue from control, pulmonary and extra-pulmonary groups of ALI mice (magnification 10X20). (B) Correlation thermograms of lung tissue samples from control, pulmonary and extra-pulmonary groups of mice. (C) Principal component analysis (PCA) plots of samples from control, pulmonary and extra-pulmonary groups.

A

Control

Cell type	Quantities	Percentage
Alveolar type I epithelial cell(AT I)	4	0.034928397
Alveolar type II epithelial cell(AT II)	11	0.096053091
B cell	4465	38.98882291
Basophil	39	0.340551869
Dendritic cell (DC)	151	1.318546979
Endothelial cell	89	0.777156829
Fibroblast	255	2.226685295
M1 macrophage	204	1.781348236
M2 macrophage	103	0.899406217
Monocyte	1231	10.74921411
Myofibroblast	93	0.812085225
Natural killer T cell (NKT)	576	5.029689137
Neutrophil	1071	9.35207824
Pericyte	40	0.349283968
Regulatory T cell (Treg)	448	3.91198044
T helper (Th) cell	2635	23.00908138
Undefined	37	0.32308767
Total number	11452	100

B

Pulmonary

Cell type	Quantities	Percentage
Alveolar type I epithelial cell(AT I)	7	0.143707658
Alveolar type II epithelial cell(AT II)	3	0.061588996
B cell	820	16.8343256
Basophil	8	0.164237323
Dendritic cell (DC)	33	0.677478957
Endothelial cell	24	0.492711969
Fibroblast	124	2.545678505
M1 macrophage	33	0.677478957
M2 macrophage	50	1.026483268
Monocyte	367	7.534387189
Myofibroblast	41	0.84171628
Natural killer T cell (NKT)	227	4.660234038
Neutrophil	2465	50.60562513
Pericyte	5	0.102648327
Regulatory T cell (Treg)	106	2.176144529
T helper (Th) cell	541	11.10654896
Undefined	17	0.349004311
Total number	4871	100

C

Extra_pulmonary

Cell type	Quantities	Percentage
Alveolar type I epithelial cell(AT I)	56	0.515843773
Alveolar type II epithelial cell(AT II)	67	0.617170228
B cell	1797	16.55305822
Basophil	37	0.34082535
Dendritic cell (DC)	292	2.689756817
Endothelial cell	55	0.506632277
Fibroblast	424	3.905674282
M1 macrophage	155	1.427781872
M2 macrophage	365	3.362196021
Monocyte	2241	20.64296242
Myofibroblast	74	0.6816507
Natural killer T cell (NKT)	757	6.973102432
Neutrophil	3024	27.85556374
Pericyte	2	0.018422992
Regulatory T cell (Treg)	245	2.256816507
T helper (Th) cell	1212	11.16433309
Undefined	53	0.488209285
Total number	10856	100

SUPPLEMENTARY FIGURE S3. Number of mouse lung tissue cells in the control (A), pulmonary (B), and extra-pulmonary groups(C) and their percentage in the groups. The number in the picture represents the true number of cells in the corresponding group of data for that group after quality control and cell sorting, and the percentage represents the percentage of cells in that group out of all cells in the corresponding group.

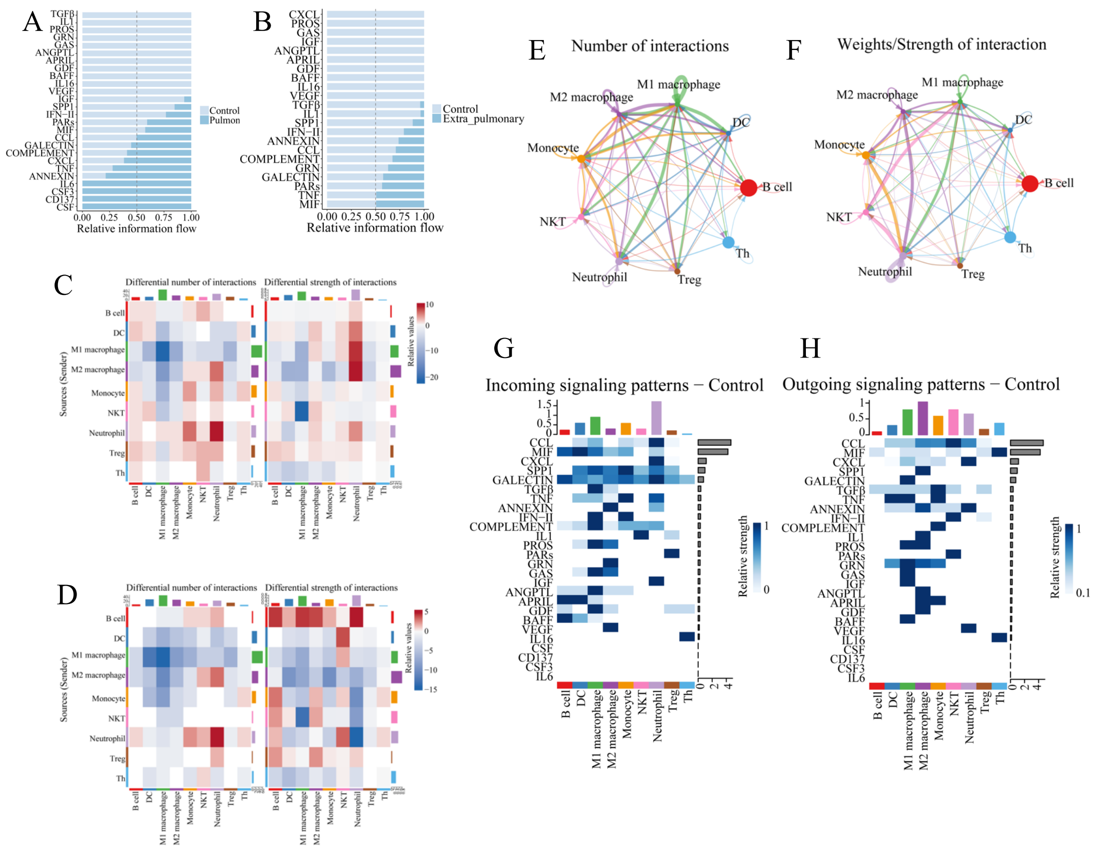

SUPPLEMENTARY FIGURE S4. The results of cellular communication in the control, pulmonary and extra-pulmonary groups showed a huge difference. (A) Bar graph of intercellular communication signals in the control and pulmonary groups. (B) Histograms of intercellular communication signals in the control and extra-pulmonary groups. (C, D) Heatmaps of changes in the number and strength of cellular communication in the pulmonary and extra-pulmonary groups compared to the control group. (E,F) Network plots of the number and intensity of cellular communications in the control group. (G,H) Heat maps of cellular input/output signals in the control group.

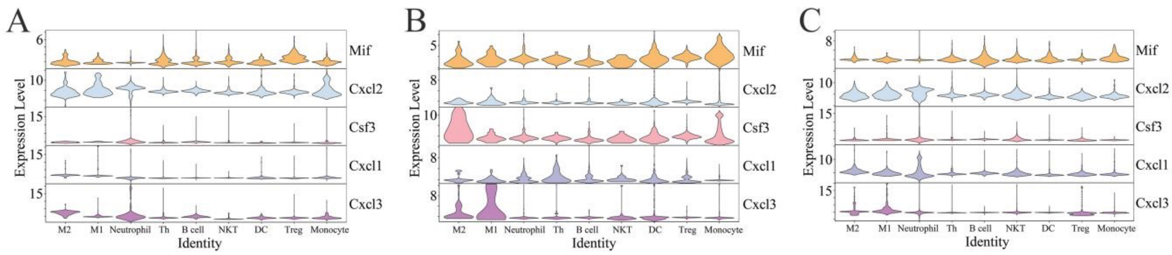

SUPPLEMENTARY FIGURE S5. Gene expression of Mif, Cxcl1, Cxcl2, Cxcl3 and Csf3 in each population of cells. (A) Expression of five genes in the control group. (B) Expression of five genes in the pulmonary group. (C) Expression of five genes in the extra-pulmonary group.

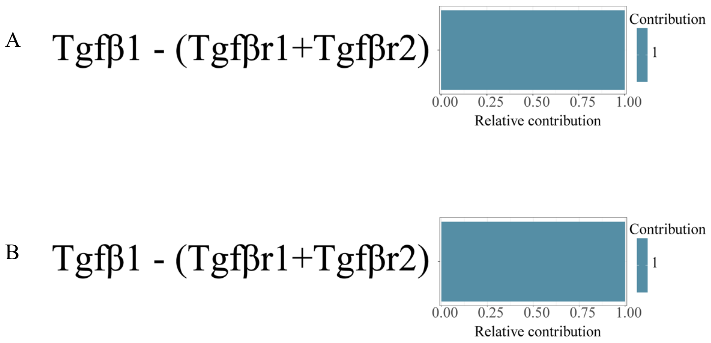

SUPPLEMENTARY FIGURE S6. Histogram of ligand receptor contributions to the TGF β signaling pathway in the control (A) and extra-pulmonary groups (B). The depth of the color and the length of the bar represent the size of the contribution.

A

Control

Cell type	Quantities	Percentage
B cell	651	6.6010951
Basophil	18	0.1825188
CD4+ T cell	2949	29.9026567
CD8+ T cell	2702	27.3980937
Dendritic cell (DC)	60	0.6083959
Erythrocyte	1794	18.1910363
Monocyte	1277	12.9486919
Neutrophil	217	2.200365
Platelet	33	0.3346177
T helper (Th) cell	82	0.8314743
Undefined	79	0.8010546
Total number	9862	100

B

COVID_ARDS

Cell type	Quantities	Percentage
B cell	6358	25.3337052
Basophil	814	3.2434155
CD4+ T cell	5909	23.5446468
CD8+ T cell	5470	21.7954337
Dendritic cell (DC)	80	0.3187632
Erythrocyte	1645	6.5545683
Monocyte	3805	15.1611746
Neutrophil	231	0.9204287
Platelet	179	0.7132327
T helper (Th) cell	537	2.139698
Undefined	69	0.2749333
Total number	25097	100

C

Sepsis_ARDS

Cell type	Quantities	Percentage
B cell	1078	11.3282892
Basophil	13	0.136612
CD4+ T cell	2919	30.6746532
CD8+ T cell	1506	15.8259773
Dendritic cell (DC)	21	0.220681
Erythrocyte	231	2.4274905
Monocyte	3168	33.2912989
Neutrophil	375	3.9407314
Platelet	164	1.7234132
T helper (Th) cell	24	0.2522068
Undefined	17	0.1786465
Total number	9516	100

SUPPLEMENTARY FIGURE S7. Number of human peripheral blood cell in the control (A), COVID-19-associated ARDS (B) and sepsis-related ARDS(C) and their percentage in the group. The number in the picture represents the true number of cells in the corresponding group of data for that group after quality control and cell sorting, and the percentage represents the percentage of cells in that group out of all cells in the corresponding group.

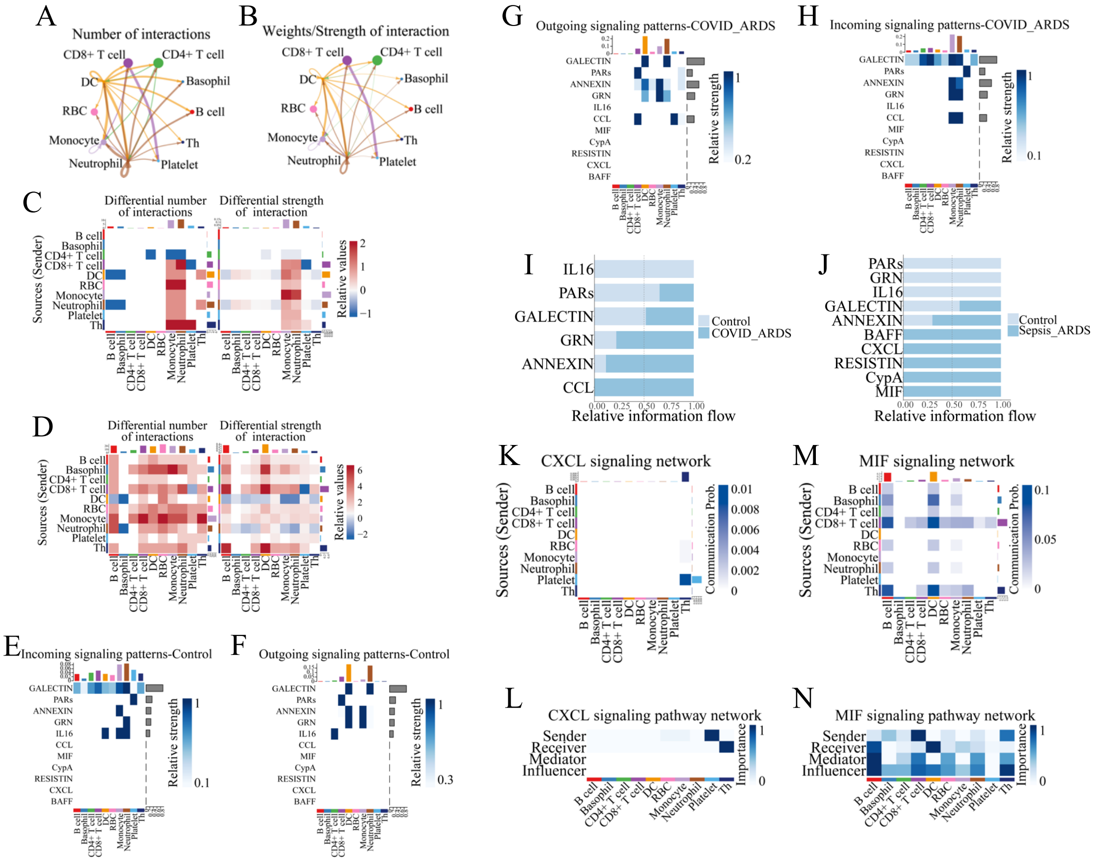

SUPPLEMENTARY FIGURE S8. Cellular communication results of control, COVID-19-associated ARDS and sepsis-related ARDS showed obvious differences. (A,B) Network map of cellular communication in the control group .(C,D) Changes in the number and strength of cellular communication in the COVID-19-associated ARDS and sepsis-related ARDS groups compared to the control group. (E-H) Heatmaps of cellular input/output signals in the control and COVID-19-associated ARDS groups. (I) Histogram of signaling pathways of cellular communication in control and COVID-19-associated ARDS groups. (J) Histogram of signaling pathways for cellular communication in the control and sepsis-related ARDS groups. (K,M) Heatmap of CXCL and MIF cellular communication in the sepsis-related ARDS group. (L,N) Heatmap of the roles assumed by cells in the CXCL and MIF signaling pathways in the sepsis-related ARDS group.
